# Conventional imaging techniques plus ^18^F-Fluorocholine PET/CT: a comparative study of diagnostic accuracy in localizing parathyroid adenomas in primary hyperparathyroidism

**DOI:** 10.3389/fendo.2025.1595461

**Published:** 2025-07-14

**Authors:** Wei-Yih Chiu, Kuen-Yuan Chen, Chia-Tung Shun, Ming-Hsun Wu, Keh-Sung Tsai, Ching-Hung Chiu, Wei-Shiung Yang, Ruoh-Fang Yen

**Affiliations:** ^1^ Division of Endocrinology and Metabolism, Department of Internal Medicine, National Taiwan University Hospital and National Taiwan University College of Medicine, Taipei, Taiwan; ^2^ Department of Laboratory Medicine, National Taiwan University Hospital, Taipei, Taiwan; ^3^ Division of General Surgery, Department of Surgery, National Taiwan University Hospital, Taipei, Taiwan; ^4^ Department of Pathology, National Taiwan University Hospital, Taipei, Taiwan; ^5^ Department of Nuclear Medicine, National Taiwan University Hospital, Taipei, Taiwan; ^6^ Graduate Institute of Clinical Medicine, College of Medicine, National Taiwan University, Taipei, Taiwan

**Keywords:** ^18^F-Fluorocholine PET/CT, neck ultrasound, technetium-99m sestamibi scintigraphy, primary hyperparathyroidism, parathyroid hormone

## Abstract

**Background:**

Currently ^18^F-Fluorocholine (FCH)-PET/CT is a choice beyond widely used techniques like ultrasound (US) and technetium-99m sestamibi (MIBI) for primary hyperparathyroidism (pHPT). It remains uncertain how FCH-PET/CT collaborates with those two traditional modalities. This study aims to prospectively evaluate the effectiveness of individual, complementary, and combined utilization of FCH-PET/CT for preoperative localization.

**Methods:**

All participants underwent US, MIBI, and FCH-PET/CT examinations, and eligible patients underwent parathyroid surgery based on surgical indications and patient preferences. McNemar’s test compared diagnostic performance between imaging techniques and Spearman’s rank correlation correlated FCH-PET/CT parameters with lesion volume, laboratory, and histological features.

**Results:**

63 out of 83 recruited patients underwent parathyroidectomy. Histologically confirmed parathyroid lesions were found in 69 glands among 63 patients. FCH-PET/CT exhibited higher sensitivity than US, MIBI, and US/MIBI combination (87.0% vs. 49.3%, *P <*0.001; vs. 49.3%, *P <*0.001; vs. 66.7%, *P*=0.006). As a second-line modality after US, MIBI, and US/MIBI combination, FCH-PET/CT achieved sensitivities of 88.6%, 77.1%, and 80.9% in detecting US-negative lesions, MIBI-negative lesions, and lesions with negative or conflicting US/MIBI results, respectively. Among various imaging combinations, the combined use of US and FCH-PET/CT showed significantly higher sensitivity than FCH-PET/CT alone (94.2% vs. 87.0%, *P*=0.025) and similar sensitivity with higher specificity than the combination of all three modalities (sensitivity: 94.2% vs. 95.7%, *P*=0.317; specificity: 98.9% vs. 95.1%, *P*=0.008).

**Conclusions:**

FCH-PET/CT is effective as a first-line or complementary technique, irrespective of prior US, MIBI or US/MIBI combination. US combined with FCH-PET/CT appears to be the most effective localization strategy among the modalities evaluated in this study. Our findings support an ultrasound-first approach for localizing primary hyperparathyroidism, with FCH-PET/CT referral in uncertain cases to enhance success rates.

## Introduction

Primary hyperparathyroidism (pHPT) is a relatively common disorder characterized by the excessive secretion of parathyroid hormone (PTH), leading to hypercalcemia, hypophosphatemia, and osteoporosis (1). With advances in parathyroid preoperative localization techniques such as neck ultrasound (US) and technetium-99m sestamibi (MIBI), minimal neck exploration has become the first choice of surgical treatment over the past decades (2, 3). While US is cost-effective and carries no ionizing radiation, its diagnostic accuracy is operator-dependent, with reported sensitivity ranging from 51% to 96% (4). MIBI scintigraphy is a well established radionuclide technique for preoperative localization, with a sensitivity from 70% to 86% (5). However, the localization accuracy of US and MIBI can be as low as <50% in the presence of multiglandular parathyroid disease, small glands or multinodular goiter (6–8). In recent years, ^18^F-Fluorocholine (FCH) positron emission tomography/computed tomography (FCH-PET/CT) has been increasingly used to detect parathyroid adenoma (9–14), offering higher sensitivity than US (9, 12) and MIBI (9–14), and is cost-effective in patients with pHPT (15–17). Nevertheless, FCH-PET/CT is much more costly than US and MIBI and it is only available at medical centers with cyclotron facilities. In clinical settings, imaging choices before surgery vary based on factors like availability, cost, and efficacy. According to the current guidelines (18), the primary imaging tools for pHPT are US and/or MIBI. However, the studies evaluating the additional sensitivity benefit of combining FCH-PET/CT with conventional modalities are scarce. The purpose of this prospective study was to assess the performance of the diverse use of FCH-PET/CT, especially when used in conjunction with or as a complement to US, MIBI, or both, in the preoperative localization of parathyroid lesions in pHPT patients.

## Materials and methods

### Study population

Patients with pHPT were included in the study, diagnosed by elevated or high normal parathyroid hormone (PTH) levels and hypercalcemia. Serum albumin, creatinine, calcium, phosphorus, 25-hydroxyvitamin D and intact-PTH were concomitantly measured. Twenty-four-hour urine calcium and creatinine while on their usual home diet were collected at baseline. The exclusion criteria were conditions known to influence parathyroid and minerals metabolism; vitamin D deficiency with serum 25-hydroxyvitamin D <10 ng/mL; renal disease with serum creatinine >2.4 mg/dL or estimated glomerular filtration rate (eGFR) <30 ml/min; the use of systemic corticosteroid, or any other treatment affecting bone metabolism within the previous 6 months; or any use of anti-resorptive agent within the previous 12 months. To evaluate complications of pHPT, history of fragility fractures and renal calculi were recorded. Bone mineral density and kidney imaging studies were used to look for bone weakness and kidney stones, respectively. The study protocol (201411046MINB) and consent document received approval from the Institutional Review Board at the author’s hospital. Informed consent was obtained from all individual participants included in the study.

### Parathyroid localization techniques: neck US, MIBI scintigraphy, and FCH-PET/CT

All patients underwent neck US, dual-phase MIBI scintigraphy and FCH-PET/CT within 4 weeks after enrollment. The sequence of imaging tests was determined by availability and was not fixed. Each examination was interpreted independently before surgery. During the US examination, color Doppler imaging and/or ultrasound-guided fine-needle aspiration cytology were performed on all detected lesions. MIBI-SPECT/CT imaging was conducted using single-photon emission computed tomography/x-ray computed tomography (SPECT/CT). Pictures of the neck and chest were taken at 20 minutes and 2 hours after the injection of 740 MBq MIBI. Images were interpreted by certified nuclear medicine specialists. FCH-PET/CT was performed after fasting for at least 6 hours. Regional PET of the neck and whole-body PET from skull base to upper thighs were started at 30 minutes and 70 minutes after the injection of 185 MBq FCH using a GE Discovery ST PET/CT Scanner (GE Medical Systems, Milwaukee, WI, USA). Transmission scans were obtained with x-ray CT without contrast agents. The images were reconstructed by iterative reconstruction with CT-derived attenuation correction using the ordered subset expectation maximization algorithm. Images were evaluated on the manufacturer’s review station (Xeleris; GE Medical System, Milwaukee, WI, USA). The maximum of standardized uptake values were measured for each lesion visualized in the early regional images (SUV1) and in the delay whole-body images (SUV2).

### Surgery, histopathologic finding and evolution of serum PTH levels

Indications for surgical intervention included those of 2022 the Fifth International Workshop in Primary Hyperparathyroidism guidelines (18). Exploration to search for abnormal parathyroid glands was performed under general anesthesia through a transverse collar incision with a retrothyroid approach. All excised glands were histologically verified. The volume of each parathyroid gland was estimated using the ellipsoid formula ([length × width × height] × π/6), where the length, width, and depth were the dimensions of gland in millimeters. The PTH serum concentrations were measured preoperatively and on the day following surgery. Success of surgery was confirmed by histopathologic findings, a decrease of more than 50% in serum PTH levels after operation, and reaching a cure, which is defined as normocalcemia for 6 months after parathyroidectomy (19).

### Performance analysis

The results of US, MIBI scintigraphy and FCH-PET/CT were evaluated, with surgical exploration and histopathological proof as the reference standard. The results of these parathyroid localization modalities were categorized as follows: true positive (TP) when a focus corresponded to an abnormal parathyroid gland in the same location, false positive (FP) when abnormal foci were detected without a corresponding abnormal parathyroid gland, and false negative (FN) when abnormal glands were missed. The true negative (TN) was the number of non-removed parathyroid glands, assuming all patients had 4 glands, when a cure from pHPT was obtained. The combined use of two or three imaging modalities was considered positive if at least one of them showed a parathyroid gland on the thyroid bed. To assess imaging performance, detection rate was calculated as the number of TP over the total number of abnormal foci on a per-patient based analysis. The parameters of diagnostic performance, including accuracy, sensitivity, specificity, positive predictive value (PPV), and negative predictive value (NPV), were calculated on a per-lesion basis.

### Sample size estimation

Based on the results of earlier studies (9–14), a pooled sensitivity of around 90% was observed with FCH PET/CT while US and MIBI both have a sensitivity of around 70%. To calculate the required sample size for testing differences in proportions for the paired-sample design (20), we considered a Type I error rate of 0.05, a Type II error rate of 0.2, sensitivities of 70% and 90% for the lower and higher tests, respectively, and a positive predictive value of 95% for the higher test given the lower test. Based on these parameters, the required sample size was 51, and to account for a 15% rate of non-assessable patients, 60 patients were targeted for enrollment overall.

### Statistical methods

Continuous variables were presented as median and interquartile range (IQR), whereas categorical variables were expressed in frequency or percentage. The rates of all outcomes were calculated with 95% confidence intervals (CIs).To compare the parameters of diagnostic performance (categorical outcomes) between these imaging techniques, Fisher’s exact test and McNemar’s Chi-square test were used for data respectively from unrelated groups and the same group. For continuous variables, Wilcoxon rank-sum test was used to compare the difference between two independent groups, while a paired sign test was used for comparison of data from the same participants. The strength and direction of association between the intensity of FCH uptake and laboratory parameters as well as adenoma size were assessed using Spearman’s rank correlation. A *P*-value less than or equal to 0.05 was considered statistically significant. All statistical analyses were conducted using Stata, version 12.1 (StataCorp, College Station, Texas, US).

## Results

**Figure 1:**
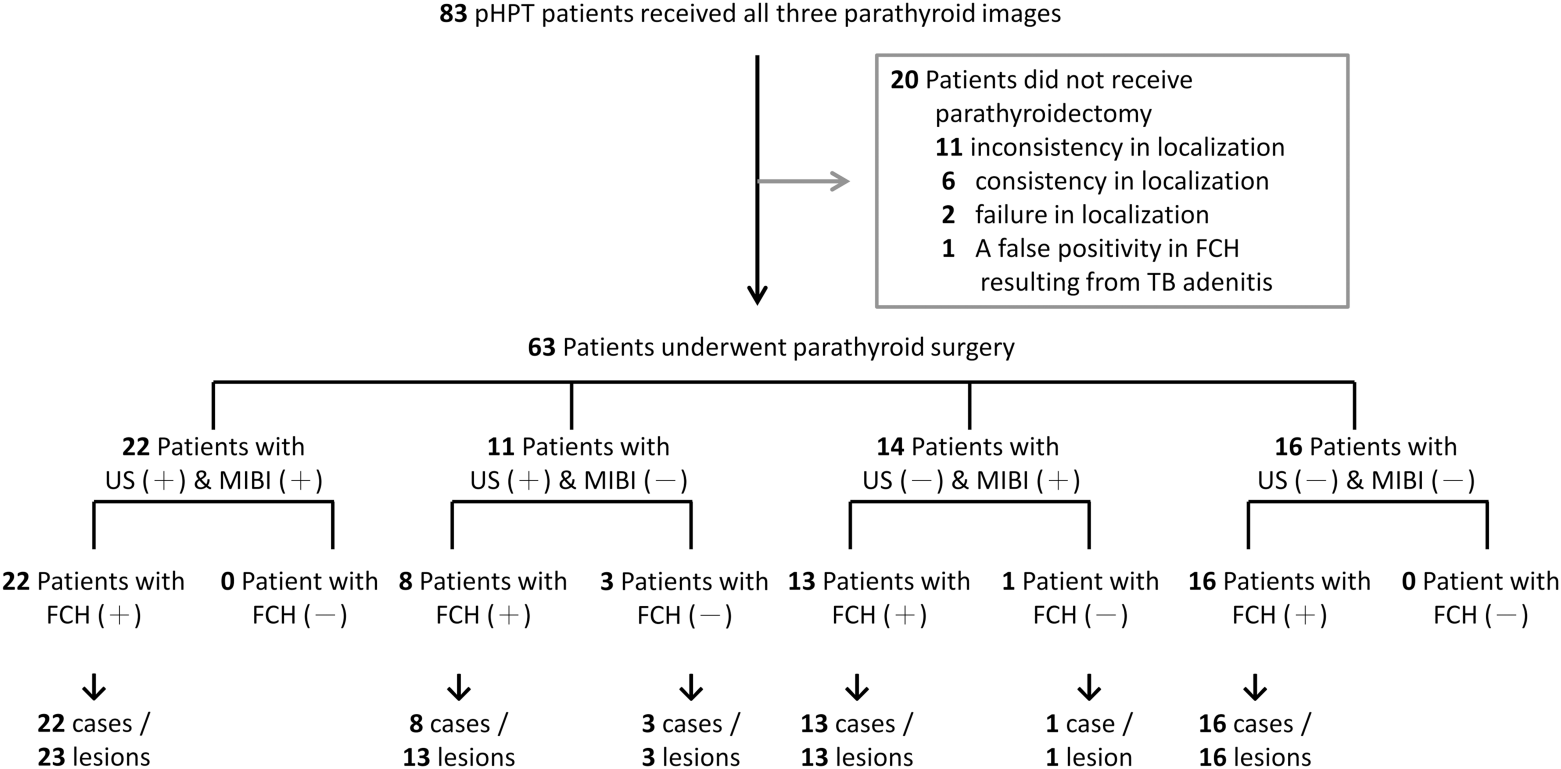
Participant flow diagram. 83 participants were recruited in this study and underwent all three parathyroid localization techniques. Twenty patients declined referral for parathyroidectomy and opted for periodic monitoring, whereas sixty-three patients underwent parathyroid surgery. Parathyroid neoplasms were confirmed in 69 lesions from these 63 patients.

We initially recruited 83 patients with symptomatic or asymptomatic pHPT (**Figure 1**). All these patients underwent neck US, MIBI-SPECT/CT and FCH-PET/CT examinations after enrollment. Surgical indications were evaluated by using the criteria updated in 2022 by the Fifth International Workshop on Primary Hyperparathyroidism (18). Among these 83 patients, 19 cases (22.9%) refused surgical referral because of failure in localization on all three modalities (2/19), personal choice based on inconsistent imaging results (11/19), or personal choice even with consistent results (6/19). One patient underwent video-assisted thoracoscopic surgery for a possible ectopic parathyroid lesion with FCH uptake in the mediastinum. Tuberculosis lymphadenitis was proven histopathologically and by tuberculosis culture. He refused further surgical intervention for persistent hyperparathyroidism with hypercalcemia. In the end, a total of 63 cases received surgical neck exploration. Histopathological examination confirmed parathyroid neoplasms in 69 glands among 63 patients. The patient characteristics are described in **Table 1**. Among the 63 patients (17 men and 46 women), the median age was 65 years, with median serum calcium at 2.74 mmol/L, phosphorus at 2.8 mg/dL, and PTH at 111 pg/mL. Of these cases, 59 (93.6%) had a single parathyroid neoplasm, 3 (4.8%) had multiglandular disease, and 1 (1.6%) had carcinoma. The median volume of the 69 surgically removed parathyroid glands was 235.6 mm^3^ (IQR, 402.1 mm^3^).

**Table 1:** Baseline characteristics of 63 patients who underwent parathyroid surgery.

Characteristic	Data^*^
Female	46 (73.0%)
Age, years	65.0 ± 14.0 (63)
BMI, kg/m2	24.2 ± 5.3 (63)
Serum albumin, g/dL	4.35 ± 0.5 (60)
Serum creatinine, mg/dL	0.8 ± 0.50 (63)
eGFR, mL/min/1.73m^2^	76.8 ± 45.4 (63)
Serum calcium, mg/dL	10.96 ± 1.0 (63)
Serum phosphorus, mg/dL	2.8 ± 0.7 (62)
Serum chloride, mEq/L	106 ± 3 (55)
Serum intact PTH, pg/mL	111 ± 86.9 (63)
Serum alkaline phosphatase, U/L	78 ± 38 (59)
25-OH Vitamin D, ng/mL	18.6 ± 11.55 (48)
Daily calcium excretion, mg	202.1 ± 156.4 (56)
Asymptomatic pHPT	9 (14.3%)
pHPT surgical indications	
Alteration in renal function (eGFR < 60 ml/min)	17 (27.0%)
serum calcium >1 mg/dl above the normal upper limit	15 (23.8%)
Presence of fragility fractures	11 (17.5%)
Osteoporosis (T-score ≦-2.5)	22 (34.9%)
Daily calcium loss >400 mg	5 (7.9%)
Age <50 years	4 (6.3%)
Presence of nephrolithiasis	21 (33.3%)

^*^Continuous variables were presented as median ± IQR (n), whereas categorical variables were expressed in frequency (percentage).

### Higher detection rates of FCH-PET/CT compared to US and MIBI scintigraphy

The comparisons of diagnostic performance among FCH-PET/CT and US, MIBI are presented in **Table 2**. On a per-patient level, the overall detection rates of US, MIBI, and FCH-PET/CT were 50.8%, 54.0%, and 93.7%, respectively. On a per-lesion level, the overall accuracy, sensitivity and specificity of US were 85.3%, 49.3% and 98.9%, and of MIBI-SPECT/CT respectively were 83.3%, 49.3% and 96.2%, and of FCH-PET/CT were individually 96.4%, 87.0%, and 100%. Furthermore, FCH-PET/CT demonstrated a significantly higher localization rate compared to US and MIBI in both patient-based and lesion-based analyses, with statistically significant differences confirmed by McNemar’s tests (*P* < 0.001).

**Table 2:** Diagnostic performance of US, MIBI-SPECT/CT, and FCH-PET/CT In 63 patients who underwent parathyroid surgery.

Tools	Per-patient based analysis			Per-lesion based analysis								
	TP	FN	DR	TP	FN	FP	TN	Accuracy	Sensitivity	Specificity	PPV	NPV
US	32	31	50.8%^***^ (37.9-63.6)	34	35	2	181	85.3%^***^ (80.3-89.4)	49.3%^***^ (37.0-61.6)	98.9%^ns^ (96.1-99.9)	94.4% (81.3-99.3)	83.8% (78.2-88.4)
MIBI	34	29	54.0%^***^ (40.9-66.6)	34	35	7	176	83.3% ^**^ (78.1-87.7)	49.3%^***^ (37.0-61.6)	96.2%^*^ (92.3-98.4)	82.9% (67.9-92.8)	83.4% (77.7-88.2)
FCH	59	4	93.7% (84.5-98.2)	60	9	0	183	96.4% (93.9-98.4)	87.0% (76.7-93.9)	100% (98.0-100)	100% (94.0-100)	95.3% (91.3-97.8)

The symbols indicated statistical significance levels between FCH-PET/CT and US or MIBI by using McNemar’s tests, e.g. ^ns^ for P ≧0.05, ^*^ for P <0.05, ^**^ for P<0.01, and ^***^ for P<0.001. Data in parentheses are 95% CIs.

DR, detection rate; FN, false negative; FP, false positive; TN, true negative; TP, true positive.

### FCH-PET/CT as the second-line in combination with the other modalities

Since FCH-PET/CT performs best among the three, we then ask whether combination with the other modalities would further improve the diagnostic performance. **Table 3** displays the performance of FCH-PET/CT as a second-line imaging method in combination with different imaging modalities. When US was employed as the first-line technique, FCH-PET/CT demonstrated a sensitivity of 88.6% and a specificity of 100% in detecting US-negative lesions. In contrast, the sensitivity and specificity of MIBI were 34.3% and 96.1%, respectively. FCH-PET/CT exhibited a significantly higher sensitivity and specificity (both *P*<0.01) compared to MIBI as a second-line method after prior US. When the combination of US and MIBI was used as the first-line technique for parathyroid imaging, the combined use of both techniques exhibited a sensitivity of 66.7% and a specificity of 95.1%. FCH-PET/CT showed significantly higher sensitivity and specificity for localizing pHPT compared to US/MIBI combination (both *P*<0.01). Notably, FCH-PET/CT successfully identified all 22 parathyroid lesions that were concordantly detected by both modalities. In cases where US and MIBI yielded negative or inconclusive results, FCH-PET/CT served as a second-line imaging modality and accurately located 38 out of 47 parathyroid adenomas. There were no instances of false positivity, resulting in a sensitivity of 80.9% and a specificity of 100.0% for FCH-PET/CT in this context.

**Table 3:** Combinations of different techniques in the preoperative localization of parathyroid lesions.

The first- and second-line tools^+^	TP	FN	FP	TN	Accuracy	Sensitivity	Specificity	PPV	NPV
(A) US → FCH									
					Performance of FCH				
Positivity on US (n=36)	29	5	0	2	86.1% (70.5-95.3)	85.3% (68.9-95.0)	100.0% (15.8-100.0)	100.0% (88.1-100)	28.6% (3.7-71.0)
Negativity on US (n=216)	31	4	0	181	98.1% (95.3-99.5)	88.6% (73.3-96.8)	100.0% (98.0-100.0)	100.0% (88.8-100.0)	97.8% (94.6-99.4)
					Performance of combined use of US and FCH				
	65	4	2	181	97.6%^**^ (94.9-99.1)	94.2%^*^ (85.8-98.4)	98.9%^ns^ (96.1-99.9)	97.0% (89.6-99.6)	97.8% (94.6-99.4)
(B) US → MIBI									
					Performance of MIBI				
Positivity on US (n=36)	22	12	0	2	66.7% (49.0-81.4)	64.7% (46.5-80.3)	100.0% (15.8-100.0)	100.0% (84.6-100.0)	14.3% (1.8-42.8)
Negativity on US (n=216)	12	23	7	174	86.1% (80.8-90.4)	34.3% (19.1-52.2)	96.1% (92.2-98.4)	63.2% (38.4-83.7)	88.3% (83.0-92.5)
					Performance of combined use of US and MIBI				
	46	23	9	174	87.3%^ns^ (82.5-91.1)	66.7%^**^ (54.3-77.6)	95.1%^**^ (90.9-97.7)	83.6% (71.2-92.2)	88.3% (83.0-92.5)
(C) MIBI → FCH									
					Performance of FCH				
Positivity on MIBI (n=41)	33	1	0	7	97.6% (87.1-99.9)	97.1% (84.7-99.9)	100% (59.0-100.0)	100% (89.4-100.0)	87.5% (47.3-99.7)
Negativity on MIBI (n=211)	27	8	0	176	96.2% (92.7-98.3)	77.1% (59.9-89.6)	100% (97.9-100)	100% (87.2-100)	95.7% (91.6-98.1)
					Performance of combined use of MIBI and FCH				
	61	8	7	176	94.0%^**^ (90.4-96.6)	88.4%^ns^ (78.4-94.9)	96.2%^**^ (92.3-98.4)	89.7% (79.9-95.8)	95.7% (91.6-98.1)
(D) US+MIBI → FCH									
					Performance of FCH				
Conclusive positivity between US and MIBI (n=22)	22	0	0	0	100.0% (84.6-100)	100.0%	—	100.0%	—
Both negativity or inclusive results between US and MIBI (n=230)	38	9	0	183	96.1% (92.7-98.2)	80.9% (66.7-90.9)	100% (98.0-100.0)	100% (90.7-100.0)	95.3% (91.3-97.8)
					Performance of pooled US+MIBI+FCH				
	66	3	9	174	95.2%^***^ (91.8-97.5)	95.7%^*^ (87.8-99.1)	95.1%^**^ (90.9-97.7)	88.0% (78.4-94.4)	98.3% (95.1-99.6)

**^+^** The left side of the rightwards arrow represented the first-line, and the right side represented the second-line.

**^#^** Statistical significance levels between FCH-PET/CT and various combinations of imaging modalities, determined by McNemar’s tests, were denoted by ^ns^ for P ≧0.05, ^*^ for P<0.05, ^**^ for P<0.01, and ^***^ for P<0.001.

Furthermore, when FCH-PET/CT is the basis of imaging technique, there was no significant improvement in sensitivity when combined with MIBI (87.0% vs. 88.4%, *P*=0.317), but a significant improvement with US (87.0% vs. 94.2%, *P*=0.025) and US/MIBI (87.0% vs. 95.7%, *P*=0.014). The specificities actually declined when combined with MIBI (100.0% vs. 96.2%, *P*=0.008) or US/MIBI (100.0% vs. 95.1%, *P*=0.003), whereas no change with US (100.0% vs. 98.9%, *P*=0.157). The combination of FCH-PET/CT and US appeared to be more effective, with a slightly higher specificity compared to the combination of all three imaging modalities (sensitivity: 94.2% vs. 95.7%, *P*=0.317; specificity: 98.9% vs. 95.1%, *P* =0.008).

### The confounding factors affecting the success rate of these parathyroid imaging modalities

To evaluate the confounding factors associated with parathyroid imaging modalities, the preoperative characteristics and postsurgical findings were compared in patients with failed versus successful results (**Supplementary Table 1**). Renal impairment was associated with a lower success rate of FCH-PET/CT. Its success rate was affected by serum creatinine level (1.3 vs. 0.8 mg/dL, *P*= 0.029) and eGFR (52.3 vs. 85.8 ml/min, *P*= 0.028). The success rate of FCH-PET/CT in patients with eGFR less than 60 ml/min was significantly lower (OR, 0.094; 95% CI, 0.009-0.983) compared to those with eGFR ≧60 ml/min. The presence of multiglandular parathyroid disease significantly influenced the localization success of FCH-PET/CT and MIBI scintigraphy. The sensitivity of FCH-PET/CT was 93.3% for parathyroid adenoma and 44.4% for multiglandular disease, with a statistical significance (*P*=0.001). Similarly, there was a significant difference in the success rate of MIBI for lesions with single- versus multiple-gland disease (55.0% vs. 11.1%, *P*=0.028). The success rate of FCH-PET/CT and MIBI in detecting single-gland disease increased by 17.5-fold (95% CI, 3.325-92.094) and 9.8-fold (95% CI, 1.150-83.120), respectively, compared to multi-gland disease. In addition, success of US and MIBI both correlated with the gland volume (141.4 vs. 367.3 mm^3^, *P* < 0.001 for US; 178.0 vs. 340.1 mm^3^, *P <*0.01 for MIBI). Furthermore, out of the 60 lesions identified as true-positives on FCH-PET/CT, the median volume of 19 lesions with false-negative results on both US and MIBI was 125.7 mm3, 19 lesions with inconsistent results between US and MIBI was 219.9 mm3, and 22 lesions with true-positive results on both US and MIBI was 388.5 mm3. There was a smaller size among glands successfully identified by FCH-PET/CT alone versus lesions correctly detected by FCH-PET/CT and either of US/MIBI (*P*=0.014), and those concordantly localized on all three imaging modalities (*P*<0.001). Our comparative analysis indicated that FCH-PET/CT outperformed conventional imaging methods, allowing for the detection of smaller lesions.

### The relationship of FCH uptake intensity with other variables

Out of 69 histologically ascertained parathyroid glands, 60 lesions were successfully detected by FCH-PET/CT. Among them, 58 lesions were visible at both imaging times, one gland only at the first imaging time, and the remaining one only at the second imaging time. Regarding the quantification of FCH uptake, the SUV1 ranged from 2.0 to 13.4, with a median of 5.2 (n=59; IQR 3.6). The SUV2 was in the range of 2.2 to 15.2, with a median of 5.3 (n=59; IQR 3.4). There was no significant difference between the SUVmax of parathyroid lesions in early and late imaging (sign test *P*=0.1112) and they were strongly correlated (r=0.799, *P <*0.001). Both SUV1 and SUV2 were positively correlated with the adenoma size (r = 0.328, *P*=0.011 for SUV1; r = 0.332, *P*=0.010 for SUV2 in the **Supplementary Table 2**). Besides, serum PTH concentration and SUV2 were positively correlated (r = 0.325, *P*=0.012). There was no significant correlation of FCH retention with cellular type (non-oxyphil cells vs. oxyphils), histological pattern (single- vs. multiple-gland disease), baseline characteristics (age, sex, body-mass index), or other preoperative serum biochemical variables such as calcium, phosphorus, alkaline-phosphatase, 25-hydroxyvitamin D (**Supplementary Table 2**).

## Discussion

Currently FCH-PET/CT is a technique of choice for preoperative localization, with reported sensitivities ranging from 82%-100% and specificities ranging from 96%-100% when used as a first-line imaging modality (9–14, 21–24). When used as a second-line imaging method, FCH-PET/CT has sensitivities of 64.3%-97.2% in cases where conventional imaging methods, US and MIBI, were negative or discordant (9, 25–28). In the present work, the sensitivity of FCH-PET/CT as the first-line imaging modality was found to be 93.7% (95% CI, 84.5%-98.2%) on a per-patient based and 87.0% (95% CI, 76.7%-93.9%) on a per-lesion based analysis. These results were consistent with previous reports (9–14, 21, 22, 29). FCH-PET/CT had a specificity of 100% (95% CI, 98.0%-100%), a negative predictive value of 95.3% (95% CI, 91.3%-97.8%), and a positive predictive value of 100% (95% CI, 94.0%-100%). The overall performance of FCH-PET/CT surpassed that of US, MIBI, and US/MIBI combination. Moreover, FCH-PET/CT as a second-line method after a prior US exhibited significantly higher sensitivity and specificity for localizing pHPT than MIBI (sensitivity, 88.6% vs. 34.3%, *P <*0.01; specificity, 100% vs. 96.1%, *P <*0.01). When US/MIBI combination was the primary imaging method, FCH-PET/CT confirmed the location with 100% success in patients with concordant results. Notably, in cases where conventional US and MIBI yielded discordant or negative results, the performance of FCH-PET/CT was favorable, with a sensitivity of 80.9% and a specificity of 100%. The sensitivity of FCH-PET/CT in individuals with uncertain US and MIBI results closely aligned with earlier study estimates, ranging from 64.3% to 97.2% (9, 25–28). However, the diagnostic performance of FCH-PET/CT in this specific context was reported in series with small case numbers and/or retrospective designs, necessitating larger prospective studies for a more robust assessment.

Having all three imaging modalities (US, MIBI, and FCH-PET/CT) for each participant enables robust intra-individual comparisons, but few studies have compared all three in a single analysis. Thanseer et al. (12) prospectively compared 3 examinations for preoperative evaluation of pHPT in 54 patients. FCH-PET/CT showed a markedly superior sensitivity (100%) compared to US (69.3%) and MIBI (76.4%). The study did not investigate the complementary effects of the imaging modalities. Another study (9) retrospectively evaluated the diagnostic performance of US, MIBI, and FCH-PET/CT in 144 pHPT patients who underwent surgery. The sensitivity of FCH-PET/CT, US, MIBI, and US/MIBI combination was 99.3%, 75.2%, 65.1%, and 89.9%, respectively. FCH-PET/CT significantly outperformed the other modalities, with strong statistical support (*P*-values < 0.001). Notably, among 72 cases with negative or equivocal US and MIBI results, FCH-PET/CT showed a high sensitivity of 97.2% in a second-line setting. In the present study, FCH-PET/CT demonstrated significantly higher sensitivity than US, MIBI, and the US/MIBI combination in detecting parathyroid lesions, with sensitivity rates of 87.0% compared to 49.3% for US (*P* < 0.001), 49.3% for MIBI (*P* < 0.001), and 66.7% for the US/MIBI combination (*P* = 0.006). As a second-line imaging modality, FCH-PET/CT was highly effective in detecting lesions that were missed by US, MIBI, or the US/MIBI combination, with sensitivities of 88.6% for US-negative lesions, 77.1% for MIBI-negative lesions, and 80.9% for lesions with negative or conflicting US/MIBI results. These studies collectively support that FCH-PET/CT can be a valuable localization tool for patients with pHPT, both as a complementary and as a first-line technique. However, its use as a first-line technique is hindered by its cost and accessibility. As a result, it is often used as a secondary imaging approach after US and MIBI, and further research is necessary to determine its potential benefits in combination with standard imaging methods.

Little is known about the utility of combining FCH-PET/CT with conventional US and MIBI in parathyroid imaging. In our cohort, the combined use of FCH-PET/CT and US showed the highest diagnostic efficacy, including the highest sensitivity (94.2%) and specificity (98.9%) among various imaging modality combinations. Our finding supports that US likely remains the initial imaging method for pHPT due to its widespread availability in general practice and local healthcare institute. In cases with uncertainties, referral for FCH-PET/CT at secondary healthcare facilities can expedite interventions and improve success rate of the operation. MIBI offers minimal additional information following the combination of US and FCH-PET/CT. Given the cost-effectiveness of FCH-PET/CT for pHPT, demonstrated by van Mossel et al. (15), Yap et al. (16) and Mamou et al. (17), MIBI’s role in pHPT localization will be restricted if FCH-PET/CT becomes standard in clinical practice. The costs of neck US, MIBI, and FCH-PET/CT in Taiwan are approximately $20, $170, and $845, respectively. Notably, FCH-PET/CT is roughly 5 times more expensive than MIBI. If neck US fails to localize the lesion, it may be more cost-effective to proceed directly with a FCH-PET/CT, rather than repeating MIBI scans 2–3 times. A thorough evaluation of its cost-effectiveness and resource availability in various healthcare settings is necessary to inform clinical decision-making and optimize patient care.

Earlier studies had shown that false-negative scans of MIBI were common in pHPT with multiglandular disease and correlated with smaller abnormal glands at surgery (6–8). In the present study, both MIBI and FCH-PET/CT were insensitive in lesions with multiple glands involvement on a per-lesion based analysis. Unlike MIBI, FCH-PET/CT could detect at least one pathological lesion in cases of multiglandular disease, although not all lesions were identified. This is consistent with the finding by Talbot et al. (30) who indicated the gland-based sensitivity of FCH-PET/CT was lower for hyperplasia than for adenoma but better than using MIBI. In terms of the effect of adenoma size on success of these imaging tools, our correlation analysis demonstrated the lesion volume significantly correlated with success of US and MIBI scan. In contrast, FCH-PET/CT enabled the identification of smaller lesions, with a threshold volume of 177.0 mm^3^ for the localization of parathyroid adenoma exclusively by FCH-PET/CT. Moreover, renal impairment was associated with failure of FCH-PET/CT in our cohort. A study by Rennick et al. (31) revealed that endogenous free choline concentrations in plasma were elevated in renal impairment patients, especially in dialysis patients. FCH-PET/CT failure in pHPT patients with chronic renal insufficiency may be attributed to several factors. The presence of endogenous choline can compete with the radiotracer for uptake, reducing the effectiveness of the scan. Additionally, impaired renal excretion of the choline-labeled radiotracer can lead to higher background activity, making it challenging to interpret the results. Although the *P*-values reached statistical significance in our subgroup analyses, these findings should be interpreted with caution due to the low case numbers of renal impairment and multiglandular disease. Further investigation with larger datasets may be necessary to confirm these findings and detect differences in these subpopulations.

## Study limitations

While there are a number of valuable findings from this research, there are some shortcomings. First, given the high sensitivity of FCH-PET/CT scan and the small sample size of this work, only a small amount of the false-negative lesions on FCH-PET/CT does not allow us to identify the connection between the cell type and the semi-quantitative FCH uptake of the parathyroid gland neoplasm. The parathyroid cell type may have an important but minor effect on FCH uptake. Further studies with larger patient series and multicenter design are needed to address the relationships between the parathyroid cell type and FCH uptake of the parathyroid gland neoplasm. Second, our study had a limitation in that it did not include multiphase computed tomography (CT) of the neck, a more advanced imaging modality. Nevertheless, a network meta-analysis (32) of 8,495 patients from 119 studies demonstrated the superiority of FCH-PET/CT, followed by the CT category, while MIBI had the highest ranking in analysis before 2009. We prioritized FCH-PET/CT, US, and MIBI in our study due to their broad availability and established clinical use in detecting parathyroid pathology. Future research may benefit from investigating the added value of combining parathyroid CT with other imaging modalities, such as FCH-PET/CT and US, to enhance diagnostic accuracy and patient outcomes. Third, this study was carried out in a specialized referral center and thus may not be reflective of patients seen in general practice and local healthcare institutes. The parathyroid lesion size in this study was smaller than reported in literature (4, 5, 33), potentially accounting for our lower success rates with US and MIBI. By contrast, FCH-PET/CT was indeed a highly sensitive tool to detect parathyroid lesions without limitation on its size, resulting in a better performance than US and MIBI. Finally, the pHPT patient population in this study was predominantly characterized by hypercalcemia. FCH-PET/CT proved effective in detecting hyperfunctioning parathyroid glands in this cohort. However, its value in other patient populations, such as normocalcemic hyperparathyroidism, metastatic parathyroid carcinoma, and reoperative surgery cases, remains uncertain and warrants further investigation in larger, multi-center studies.

## Conclusion

**Figure 2:**
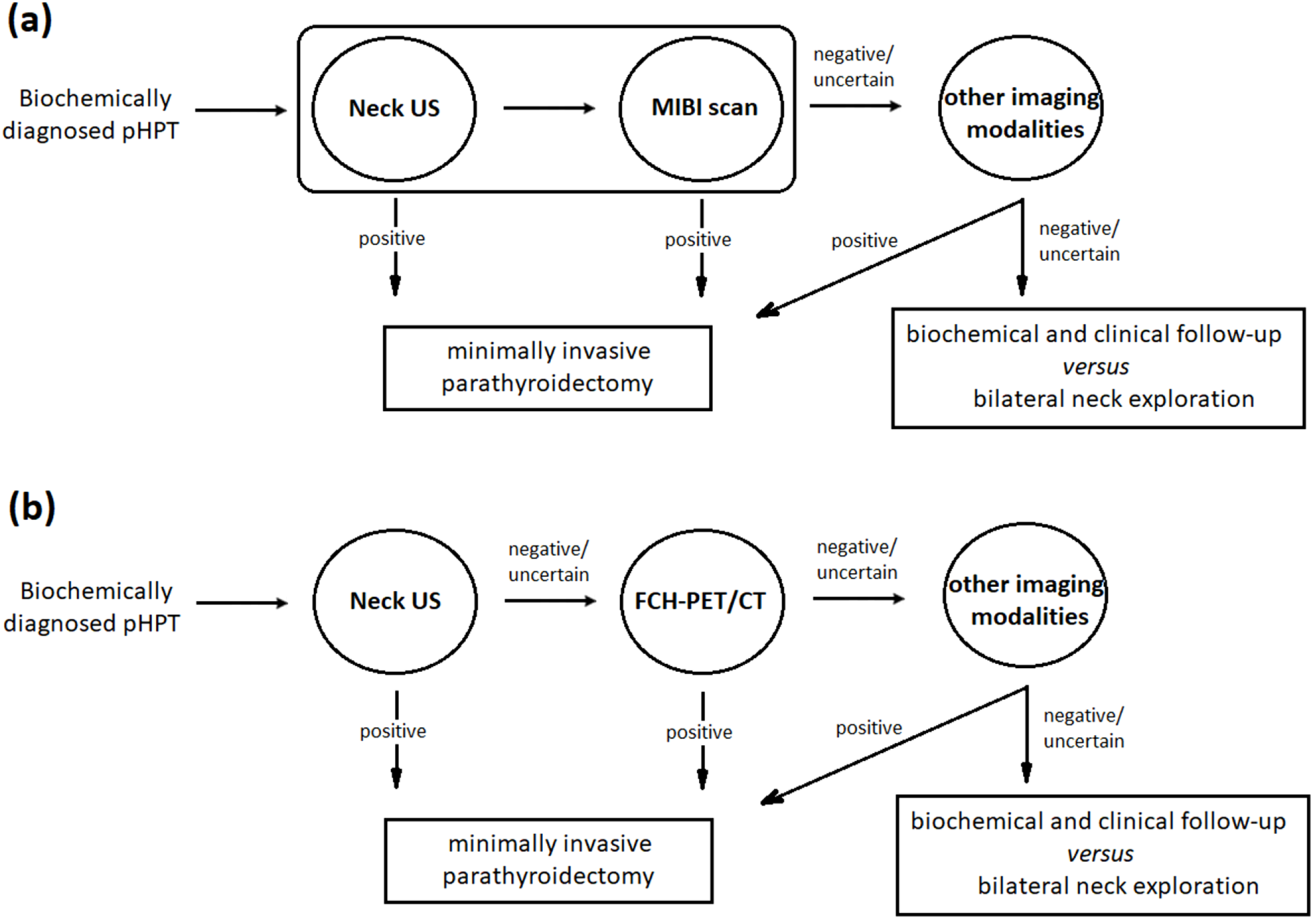
Clinical decision flowchart. In the current approach **(a)**, minimally invasive parathyroidectomy has become the preferred surgical treatment option following successful localization using US and MIBI. Other imaging modalities, such as 4D-CT, MRI, PET, and venous sampling, are typically reserved for cases with negative or inconclusive results from traditional methods. The choice of these methods depends on their cost-effectiveness and resource availability. In contrast, the proposed US/FCH-PET/CT combined strategy **(b)** supports using US as the primary modality, reserving FCH-PET/CT for cases where US results are inconclusive. Under this approach, the additional value of MIBI is minimal.

The high accuracy of FCH-PET/CT made it both a preferred choice and a valuable complementary tool in the diagnostic pathway. Gland volume significantly influenced the success of US and MIBI localization, but not FCH-PET/CT. The intensity of FCH accumulation correlated with gland volume, but not cell type or histological pattern. The combination of US and FCH-PET/CT demonstrates the highest diagnostic performance among evaluated imaging methods. This combination is highly relevant to current diagnostic practices (**Figure 2**), supporting the use of US as the primary modality, with FCH-PET/CT reserved for cases with negative or equivocal US results. When US or FCH-PET/CT results are clearly positive, the additional value of MIBI is minimal.

## Data Availability

The original contributions presented in the study are included in the article/**Supplementary Material**. Further inquiries can be directed to the corresponding authors.

## References

[1] Marcocci C Cetani F. Clinical practice. Primary hyperparathyroidism. New Engl J Med. (2011) 365:2389–97. doi: 10.1056/NEJMcp1106636, PMID: 22187986

[2] Noureldine SI Gooi Z Tufano RP. Minimally invasive parathyroid surgery. Gland Surg. (2015) 4:410–9. doi: 10.3978/j.issn.2227-684X.2015.03.07, PMID: 26425454 PMC4561661

[3] Ahmadieh H Kreidieh O Akl EA El-Hajj Fuleihan G. Minimally invasive parathyroidectomy guided by intraoperative parathyroid hormone monitoring (IOPTH) and preoperative imaging versus bilateral neck exploration for primary hyperparathyroidism in adults. Cochrane Database Syst Rev. (2020) 10:CD010787. doi: 10.1002/14651858.CD010787.pub2, PMID: 33085088 PMC8094219

[4] Mihai R Simon D Hellman P. Imaging for primary hyperparathyroidism–an evidence-based analysis. Langenbecks Arch Surg. (2009) 394:765–84. doi: 10.1007/s00423-009-0534-4, PMID: 19590890

[5] Wong KK Fig LM Gross MD Dwamena BA. Parathyroid adenoma localization with ^99m^Tc-sestamibi SPECT/CT: A meta-analysis. Nucl Med Commun. (2015) 36:363–75. doi: 10.1097/MNM.0000000000000262, PMID: 25642803

[6] Ruda JM Hollenbeak CS Stack BC Jr. A systematic review of the diagnosis and treatment of primary hyperparathyroidism from 1995 to 2003. Otolaryngology–head Neck Surg. (2005) 132:359–72. doi: 10.1016/j.otohns.2004.10.005, PMID: 15746845

[7] Milas M Wagner K Easley KA Siperstein A Weber CJ. Double adenomas revisited: nonuniform distribution favors enlarged superior parathyroids (Fourth pouch disease). Surgery. (2003) 134:995–1003. doi: 10.1016/j.surg.2003.07.009, PMID: 14668733

[8] Biertho LD Kim C Wu HS Unger P Inabnet WB. Relationship between sestamibi uptake, parathyroid hormone assay, and nuclear morphology in primary hyperparathyroidism. J Am Coll Surgeons. (2004) 199:229–33. doi: 10.1016/j.jamcollsurg.2004.04.013, PMID: 15275878

[9] Boudousq V Guignard N Gilly O Chambert B Mamou A Moranne O. Diagnostic performance of cervical ultrasound, ^99m^Tc-sestamibi scintigraphy, and contrast-enhanced ^18^F-fluorocholine PET in primary hyperparathyroidism. J Nucl Med. (2022) 63:1081–6. doi: 10.2967/jnumed.121.261900, PMID: 34857659

[10] Cuderman A Senica K Rep S Hocevar M Kocjan T Sever MJ. ^18^F-fluorocholine PET/CT in primary hyperparathyroidism: superior diagnostic performance to conventional scintigraphic imaging for localization of hyperfunctioning parathyroid glands. J Nucl Med. (2020) 61:577–83. doi: 10.2967/jnumed.119.229914, PMID: 31562221

[11] Ferrari C Santo G Mammucci P Pisani AR Sardaro A Rubini G. Diagnostic value of choline PET in the preoperative localization of hyperfunctioning parathyroid gland(S): A comprehensive overview. Biomedicines. (2021) 9:231. doi: 10.3390/biomedicines9030231, PMID: 33669104 PMC7996619

[12] Thanseer N Bhadada SK Sood A Mittal BR Behera A Gorla AKR. Comparative effectiveness of ultrasonography, ^99m^Tc-sestamibi, and ^18^F-fluorocholine PET/CT in detecting parathyroid adenomas in patients with primary hyperparathyroidism. Clin Nucl Med. (2017) 42:e491–e7. doi: 10.1097/RLU.0000000000001845, PMID: 28902729

[13] Lezaic L Rep S Sever MJ Kocjan T Hocevar M Fettich J. ^18^F-fluorocholine PET/CT for localization of hyperfunctioning parathyroid tissue in primary hyperparathyroidism: A pilot study. Eur J Nucl Med Mol Imaging. (2014) 41:2083–9. doi: 10.1007/s00259-014-2837-0, PMID: 25063039

[14] Beheshti M Hehenwarter L Paymani Z Rendl G Imamovic L Rettenbacher R. ^18^F-fluorocholine PET/CT in the assessment of primary hyperparathyroidism compared with ^99m^Tc-MIBI or ^99m^Tc-tetrofosmin SPECT/CT: A prospective dual-centre study in 100 patients. Eur J Nucl Med Mol Imaging. (2018) 45:1762–71. doi: 10.1007/s00259-018-3980-9, PMID: 29516131 PMC6097754

[15] van Mossel S Saing S Appelman-Dijkstra N Quak E Schepers A Smit F. Cost-effectiveness of one-stop-shop [^18^F]Fluorocholine PET/CT to localise parathyroid adenomas in patients suffering from primary hyperparathyroidism. Eur J Nucl Med Mol Imaging. (2024) 51:3585–95. doi: 10.1007/s00259-024-06771-1, PMID: 38837058 PMC11457719

[16] Yap A Hope TA Graves CE Kluijfhout W Shen WT Gosnell JE. A cost-utility analysis of ^18^F-fluorocholine-positron emission tomography imaging for localizing primary hyperparathyroidism in the United States. Surgery. (2022) 171:55–62. doi: 10.1016/j.surg.2021.03.075, PMID: 34340823

[17] Mamou A Chkair S Gilly O Maimoun L Mamou Y Sheppard SC. Economic evaluation of [^18^F]Fluorocholine PET/CT in pre operative assessment of hyperfunctional parathyroids in primary hyperparathyroidism: A cost effectiveness analysis. EJNMMI Rep. (2025) 9:11. doi: 10.1186/s41824-025-00244-w, PMID: 40164868 PMC11958853

[18] Bilezikian JP Khan AA Silverberg SJ Fuleihan GE Marcocci C Minisola S. Evaluation and management of primary hyperparathyroidism: summary statement and guidelines from the fifth international workshop. J Bone Miner Res. (2022) 37:2293–314. doi: 10.1002/jbmr.4677, PMID: 36245251

[19] Wilhelm SM Wang TS Ruan DT Lee JA Asa SL Duh QY. The american association of endocrine surgeons guidelines for definitive management of primary hyperparathyroidism. JAMA Surg. (2016) 151:959–68. doi: 10.1001/jamasurg.2016.2310, PMID: 27532368

[20] Connor RJ. Sample size for testing differences in proportions for the paired-sample design. Biometrics. (1987) 43:207–11. doi: 10.2307/2531961, PMID: 3567305

[21] Abhishek B Wakankar R Dharmashaktu Y Damle NA Kumar P Bal C. Comparison of neck ultrasonography, dual phase ^99m^Tc-sestamibi with early SPECT-CT & ^18^F-fluorocholine PET-CT as first line imaging in patients with primary hyperparathyroidism. Indian J Nucl Med. (2023) 38:208–17. doi: 10.4103/ijnm.ijnm_28_22, PMID: 38046978 PMC10693368

[22] Quak E Lasne-Cardon A Cavarec M Lireux B Bastit V Roudaut N. F18-choline PET/CT or MIBI SPECT/CT in the surgical management of primary hyperparathyroidism: A diagnostic randomized clinical trial. JAMA Otolaryngol Head Neck Surg. (2024) 150:658–65. doi: 10.1001/jamaoto.2024.1421, PMID: 38900416 PMC11190825

[23] Treglia G Piccardo A Imperiale A Strobel K Kaufmann PA Prior JO. Diagnostic performance of choline PET for detection of hyperfunctioning parathyroid glands in hyperparathyroidism: A systematic review and meta-analysis. Eur J Nucl Med Mol Imaging. (2019) 46:751–65. doi: 10.1007/s00259-018-4123-z, PMID: 30094461

[24] Garnier S Maheo C Potard G Cavarec MB Roudaut N Thuillier P. Contribution of ^18^F-fluorocholine PET-CT to the preoperative localisation of parathyroid adenoma for the treatment of primary hyperparathyroidism. Sci Rep. (2025) 15:10018. doi: 10.1038/s41598-025-94735-2, PMID: 40122914 PMC11930921

[25] Quak E Blanchard D Houdu B Le Roux Y Ciappuccini R Lireux B. F18-choline PET/CT guided surgery in primary hyperparathyroidism when ultrasound and MIBI SPECT/CT are negative or inconclusive: the APACH1 study. Eur J Nucl Med Mol Imaging. (2018) 45:658–66. doi: 10.1007/s00259-017-3911-1, PMID: 29270788 PMC5829113

[26] Uslu-Besli L Sonmezoglu K Teksoz S Akgun E Karayel E Pehlivanoglu H. Performance of F-18 fluorocholine PET/CT for detection of hyperfunctioning parathyroid tissue in patients with elevated parathyroid hormone levels and negative or discrepant results in conventional imaging. Korean J Radiol. (2020) 21:236–47. doi: 10.3348/kjr.2019.0268, PMID: 31997599 PMC6992441

[27] Koumakis E Gauthe M Martinino A Sindayigaya R Delbot T Wartski M. FCH-PET/CT in primary hyperparathyroidism with discordant/negative MIBI scintigraphy and ultrasonography. J Clin Endocrinol Metab. (2023) 108:1958–67. doi: 10.1210/clinem/dgad073, PMID: 36750257

[28] Michaud L Burgess A Huchet V Lefevre M Tassart M Ohnona J. Is ^18^F-fluorocholine-positron emission tomography/computerized tomography a new imaging tool for detecting hyperfunctioning parathyroid glands in primary or secondary hyperparathyroidism? J Clin Endocrinol Metab. (2014) 99:4531–6. doi: 10.1210/jc.2014-2821, PMID: 25215560

[29] Broos WAM van der Zant FM Knol RJJ Wondergem M. Choline PET/CT in parathyroid imaging: A systematic review. Nucl Med Commun. (2019) 40:96–105. doi: 10.1097/MNM.0000000000000952, PMID: 30444749

[30] Talbot JN Perie S Tassart M Delbot T Aveline C Zhang-Yin J. ^18^F-fluorocholine PET/CT detects parathyroid gland hyperplasia as well as adenoma: 401 PET/CTs in one center. Q J Nucl Med Mol Imaging. (2023) 67:96–113. doi: 10.23736/S1824-4785.23.03513-6, PMID: 36995286

[31] Rennick B Acara M Hysert P Mookerjee B. Choline loss during hemodialysis: homeostatic control of plasma choline concentrations. Kidney Int. (1976) 10:329–35. doi: 10.1038/ki.1976.116, PMID: 1032897

[32] Lee SW Shim SR Jeong SY Kim SJ. Direct comparison of preoperative imaging modalities for localization of primary hyperparathyroidism: A systematic review and network meta-analysis. JAMA Otolaryngol Head Neck Surg. (2021) 147:692–706. doi: 10.1001/jamaoto.2021.0915, PMID: 34081083 PMC8176390

[33] Alharbi AA Alshehri FM Albatly AA Sah BR Schmid C Huber GF. ^18^F]Fluorocholine uptake of parathyroid adenoma is correlated with parathyroid hormone level. Mol Imaging Biol. (2018) 20:857–67. doi: 10.1007/s11307-018-1179-x, PMID: 29508264

