# Supplementary material for: Conventional imaging techniques plus 18F-Fluorocholine PET/CT: a comparative study of diagnostic accuracy in localizing parathyroid adenomas in primary hyperparathyroidism
Source: Front Endocrinol (Lausanne). 2025 Jul 14;16:1595461. doi: 10.3389/fendo.2025.1595461 (PMC12301907; doi:10.3389/fendo.2025.1595461)
Supplement: Supplementary file 1 [file Table1.docx]

**Supplemental Table 1**

| **Supplemental Table 1. Factors affect the success rate of parathyroid localization modalities** | | | | | | | | | | | | |
| --- | --- | --- | --- | --- | --- | --- | --- | --- | --- | --- | --- | --- |
|  |  |  | **US** |  |  |  | **MIBI** |  |  |  | **FCH** |  |
| Characteristic^+^ |  | **Fail** | **Success** | *P*^#^ |  | **Fail** | **Success** | *P*^#^ |  | **Fail** | **Success** | *P*^#^ |
| Age, years |  | 63.4 ± 9.6 (31) | 66.5 ± 14.9 (32) | 0.518 |  | 64.3 ± 13.1 (29) | 65.3 ± 15.1 (34) | 0.799 |  | 61.6 ± 9.2 (4) | 65.2 ± 14.4 (59) | 0.284 |
| BMI, kg/m2 |  | 24.3 ± 6.3 (31) | 22.5 ± 4.9 (32) | 0.945 |  | 24.9 ± 5.7 (29) | 22.3 ± 4.1 (34) | 0.195 |  | 26.6 ± 8.2 (4) | 23.9 ± 5.3 (59) | 0.167 |
| Serum albumin, g/dL |  | 4.4 ± 0.5 (31) | 4.3 ± 0.5 (29) | 0.309 |  | 4.4 ± 0.55 (28) | 4.3 ± 0.45 (32) | 0.634 |  | 4.3 ± 1.0 (3) | 4.4 ± 0.5 (57) | 0.733 |
| Serum creatinine, mg/dL |  | 0.7 ± 0.4 (31) | 0.8 ± 0.6 (32) | 0.077 |  | 0.9 ± 0.5 (29) | 0.8 ± 0.4 (34) | 0.404 |  | 1.3 ± 0.7 (4) | 0.8 ± 0.4 (59) | 0.029 |
| eGFR, mL/min/1.73m^2^ |  | 89.9 ± 43.0 (31) | 75.9 ± 34.6 (32) | 0.070 |  | 71.0 ± 45.6 (29) | 86.3 ± 33.2 (34) | 0.321 |  | 52.3 ± 21.4 (4) | 85.8 ± 43.6 (59) | 0.028 |
| Serum calcium, mg/dL |  | 10.84 ± 0.84 (31) | 11.12 ± 1.08 (32) | 0.191 |  | 11.04 ± 0.96 (29) | 10.84 ± 0.68 (34) | 0.392 |  | 11.2 ± 1.68 (4) | 10.96 ± 0.88 (59) | 0.789 |
| Serum phosphorus, mg/dL |  | 2.8 ± 0.8 (31) | 2.8 ± 0.7 (31) | 0.756 |  | 2.9 ± 0.8 (29) | 2.8 ± 0.6 (33) | 0.832 |  | 2.8 ± 0.5 (4) | 2.8 ± 0.8 (58) | 0.421 |
| Serum chloride, mEq/L |  | 106 ± 3 (26) | 105 ± 4 (29) | 0.659 |  | 106 ± 4 (26) | 105 ± 3 (29) | 0.993 |  | 106 ± 7 (3) | 105.5 ± 3.5 (52) | 0.225 |
| Serum intact PTH, pg/mL |  | 101 ± 65 (31) | 124 ± 103 (32) | 0.078 |  | 117 ± 110.3 (29) | 108.5 ± 62.4 (34) | 0.815 |  | 193 ± 187 (4) | 106 ± 80 (59) | 0.076 |
| Serum alkaline phosphatase, U/L |  | 70 ± 45 (31) | 79.5 ± 30.5 (28) | 0.412 |  | 81 ± 36 (27) | 78 ± 33 (32) | 0.543 |  | 66.5 ± 47.5 (4) | 78 ± 38 (55) | 0.526 |
| 25-OH Vitamin D, ng/mL |  | 17.5 ± 11.7 (28) | 19.3 ± 10.9 (20) | 0.645 |  | 16.8 ± 13.2 (22) | 19 ± 9 (26) | 0.218 |  | 18.2 ± 0 (1) | 19 ± 12 (47) | 0.942 |
| Daily calcium excretion, mg |  | 198.1 ± 181.1 (30) | 215.3 ± 148.1 (26) | 0.366 |  | 192.2 ± 208.6 (26) | 221.5 ± 115.9 (30) | 0.264 |  | 163.6 ± 47.6 (3) | 205.6 ± 164.7 (53) | 0.413 |
| Gland volume, mm^3^ |  | 141.4 ± 188.5 (35) | 367.3 ± 439.8 (34) | <0.001 |  | 178.0 ± 234.6 (35) | 340.1 ± 445.1 (34) | 0.003 |  | 183.3 ± 139.3 (9) | 253.9 ± 426.5 (60) | 0.092 |
| Sex (female vs. male) |  | 24 (77.4%) vs. 7 (22.6%) | 22 (68.8%) vs. 10 (31.3%) | 0.572 |  | 21 (72.4%) vs. 8 (27.6%) | 25 (73.5%) vs. 9 (26.5% | 1.000 |  | 2 (50.0%) vs. 2 (50.0%) | 44 (74.6%) vs. 15 (25.4%) | 0.293 |
| Alteration in renal function (eGFR ≥60 vs. <60 ml/min) |  | 25 (80.7%) vs. 6 (19.4%) | 22 (68.8%) vs. 10 (31.3%) | 0.387 |  | 20 (69.0%) vs. 9 (31.0%) | 27 (79.4%) vs. 7 (20.6%) | 0.394 |  | 1 (25.0%) vs. 3 (75.0%) | 46 (78.0%) vs. 13 (22.0 %) | 0.047 |
| Thyroid nodule ( >1 cm) at the lesion side (absence vs. presence) |  | 16 (51.6%) vs. 15 (48.4 %) | 23 (69.7%) vs. 10 (30.3 %) | 0.200 |  | 17 (56.7%) vs. 13 (43.3 %) | 22 (64.7%) vs. 12 (35.3 %) | 0.610 |  | 2 (50.0 %) vs. 2 (50.0 %) | 37 (61.7%) vs. 23 (38.3 %) | 0.640 |
| Histological pattern (SGD vs. MGD) |  | 31 (88.6 %) vs. 4 (11.4 %) | 29 (85.3 %) vs. 5 (14.7 %) | 0.734 |  | 27 (77.1 %) vs. 8 (22.9 %) | 33 (97.1 %) vs. 1 (2.9 %) | 0.028 |  | 4 (44.4 %) vs. 5 (55.6 %) | 56 (93.3 %) vs. 4 (6.7 %) | 0.001 |
| Parathyroid oxyphils in histology (absence vs. presence) |  | 22 (71.0%) vs. 9 (29.0 %) | 22 (66.7%) vs. 11 (33.3 %) | 0.791 |  | 19 (63.3%) vs. 11 (36.7 %) | 25 (73.5%) vs. 9 (26.5 %) | 0.427 |  | 4 (100%) vs. 0 (0%) | 40 (66.7%) vs. 20 (33.3 %) | 0.300 |
| ^+^Demographic and histopathologic variables were presented on a per-patient and a per-lesion basis, respectively. Continuous variables were presented as median ± IQR (n), whereas categorical variables were expressed in frequency (percentage). | | | | | | | | | | | | |
| ^#^ Fisher’s exact test was used in the analysis of contingency tables, while Wilcoxon rank-sum test was used to determine the equality of medians in two independent samples. | | | | | | | | | | | | |

**Supplemental Table 2**

| **Supplemental Table 2. Correlation of FCH uptake with biochemical features, adenoma size, and histological findings** | | | | | | |
| --- | --- | --- | --- | --- | --- | --- |
|  |  | **SUV1** |  |  | **SUV2** |  |
| Characteristic^+^ |  | Correlation^#^ | *P*-value^#^ |  | Correlation^#^ | *P*-value^#^ |
| Age, years |  | 0.064 (59) | 0.631 |  | -0.065 (59) | 0.623 |
| BMI, kg/m2 |  | -0.011 (59) | 0.933 |  | 0.047 (59) | 0.722 |
| Serum albumin, g/dL |  | -0.167 (57) | 0.213 |  | -0.160 (57) | 0.234 |
| Serum creatinine, mg/dL |  | 0.007 (59) | 0.958 |  | 0.024 (59) | 0.856 |
| eGFR, mL/min/1.73m^2^ |  | -0.048 (59) | 0.717 |  | 0.006 (59) | 0.966 |
| Serum calcium, mg/dL |  | 0.120 (59) | 0.367 |  | 0.078 (59) | 0.557 |
| Serum phosphorus, mg/dL |  | -0.090 (59) | 0.503 |  | -0.124 (58) | 0.356 |
| Serum chloride, mEq/L |  | -0.010 (52) | 0.943 |  | 0.107 (52) | 0.450 |
| Serum intact PTH, pg/mL |  | 0.216 (59) | 0.101 |  | 0.325 (59) | 0.012 |
| Serum alkaline phosphatase, U/L |  | 0.224 (54) | 0.104 |  | 0.235 (55) | 0.084 |
| 25-OH Vitamin D, ng/mL |  | 0.142 (47) | 0.341 |  | -0.033 (47) | 0.825 |
| Daily calcium excretion, mg |  | 0.238 (53) | 0.087 |  | 0.212 (53) | 0.114 |
| Gland volume, mm^3^ |  | 0.328 (59) | 0.011 |  | 0.332 (59) | 0.010 |
| Sex (female vs. male) |  | 5 ± 3.6 (43) vs. 5.4 ± 2.8 (15) | 0.894 |  | 4.4 ± 3.3 (43) vs. 6.5 ± 3.3 (15) | 0.138 |
| Alteration in renal function (eGFR ≥60 vs. <60 ml/min) |  | 4.9 ± 2.8 (45) vs. 5.9 ± 2.8 (13) | 0.401 |  | 4.9 ± 2.9 (45) vs. 6.6 ± 4.6 (13) | 0.176 |
| Thyroid nodule ( >1 cm) at the lesion side (absence vs. presence) |  | 5.0 ± 2.6 (37) vs.  5.75 ± 3.8 (22) | 0.839 |  | 5.3 ± 3.35 (36) vs. 5.2 ± 3.4 (23) | 0.798 |
| Histological pattern (SGD vs. MGD) |  | 5.0 ± 2.8 (55) vs. 7.7 ± 3.85 (4) | 0.113 |  | 5.2 ± 3.3 (55) vs. 8.25 ± 5.7 (4) | 0.240 |
| Parathyroid oxyphils in histology (absence vs. presence) |  | 4.85 ± 2.8 (40) vs. 6.0 ± 3.6 (19) | 0.117 |  | 5.7 ± 3.6 (39) vs. 5.0 ± 3.15 (20) | 0.773 |
| ^+^Demographic and histopathologic variables were presented on a per-patient and a per-lesion basis, respectively. Correlation results are presented as the correlation coefficient (n) or median ± IQR (n). | | | | | | |
| ^#^Statistical tests were conducted using Spearman’s rank correlation or Wilcoxon rank-sum tests. | | | | | | |
